# Transcriptomic analysis of pancreatic cancer cells in response to metformin and aspirin: an implication of synergy

**DOI:** 10.1038/srep13390

**Published:** 2015-08-21

**Authors:** Wen Yue, Tao Wang, Emmanuel Zachariah, Yong Lin, Chung S. Yang, Qing Xu, Robert S. DiPaola, Xiang-Lin Tan

**Affiliations:** 1 Rutgers Cancer Institute of New Jersey, Rutgers, The State University of New Jersey, New Brunswick, NJ 08901; 2 Department of Epidemiology & Population Health, Albert Einstein College of Medicine, Bronx, NY 10461; 3 Department of Biostatistics, School of Public Health, Rutgers, The State University of New Jersey, Piscataway, NJ 08854; 4 Department of Chemical Biology, Ernest Mario School of Pharmacy, Rutgers, The State University of New Jersey, Piscataway, NJ 08854; 5 Department of Oncology, Shanghai Tenth People’s Hospital, Tongji University, School of Medicine, Shanghai, 200072; 6 Department of Epidemiology, School of Public Health, Rutgers, The State University of New Jersey, Piscataway, NJ 08854.

## Abstract

Metformin and aspirin have been studied extensively as cancer preventative and therapeutic agents. However, the underlying molecular mechanisms for the inhibitory effects of pancreatic cancer development remain undefined. To gain further insight into their biological function in pancreatic cancer, we conducted a transcriptomic analysis using RNA sequencing to assess the differential gene expression induced by metformin (5 mM) and aspirin (2 mM), alone or in combination, after treatment of PANC-1 cells for 48 hours. Compared to an untreated control, metformin down-regulated 58 genes and up-regulated 91 genes, aspirin down-regulated 12 genes only, while metformin plus aspirin down-regulated 656 genes and up-regulated 449 genes (fold-change > 2, *P* < 10^−5^). Of the top 10 genes (fold-change > 10, *P* < 10^−10^) regulated by metformin plus aspirin, *PCDH18, CCL2, RASL11A, FAM111B* and *BMP5* were down-regulated ≥ 20-fold, while *NGFR, NPTX1*, C7orf57, MRPL23AS1 and *UNC5B* were up-regulated ≥ 10-fold. Ingenuity Pathway Analysis (IPA) revealed that the pathways, “cholesterol biosynthesis”, “cell cycle: G1/S checkpoint regulation”, and “axonal guidance signaling” were the most statistically significant pathways modulated by metformin plus aspirin. Although the results need further functional validation, these data provide the first evidence for the synergistic action between metformin and aspirin in modulating the transcriptional profile of pancreatic cancer cells.

Pancreatic cancer is the fourth leading cause of cancer-related death, which accounts for more than 39,000 annual cancer deaths in the United States. Moreover, the overall 5-year survival rate of pancreatic cancer is still less than 5% indicating poor prognosis^1^. Currently, surgery, chemotherapy, and radiotherapy are main treatment approaches for pancreatic cancer. However, the mortality rate remains very high because of the low rate of surgical patients and non-sensitivity to chemotherapy and radiotherapy. Therefore, to explore new strategies of prevention and treatment for pancreatic cancer is urgent and important. Metformin and aspirin are emerging as cancer prevention candidate drugs in recent years, and numerous experimental data have demonstrated that both metformin and aspirin have a direct anti-proliferative effect on pancreatic cancer cells *in vitro* and *in vivo*^2^. However, the underlying molecular mechanisms for the inhibitory actions of pancreatic cancer progression remain largely unknown.

Potential mechanisms of metformin’s anti-neoplastic effect include the activation of 5′ AMP-activated protein kinase (AMPK)^3^, inhibition of mTOR pathway^4^, and lowering hyperinsulinemia^5^, modulating inflammatory responses^6^, as well as selectively killing cancer stem cells^7^. Aspirin with potential effects on AMPK-mTOR signaling^8^,^9^ also modulates multiple inflammatory components, e.g. COX1/COX2, nuclear factor kappa B (NFкB)^10^. Additionally, both metformin and aspirin have been reported to inhibit signal transducer and activator of transcription 3 (STAT3)^11^,^12^. These findings indicate that both metformin and aspirin are multi-target drugs that exert the anti-cancer effect through simultaneously targeting various signaling pathways and molecules that are involved in tumor initiation and development. The crosstalk and interaction among different signaling pathways is critical for understanding the underlying anti-cancer mechanism of metformin and aspirin action. However, an overview of genes and pathways related to metformin and aspirin action remains relatively understudied. Therefore, it is important to obtain a comprehensive profile of genes and pathways that are regulated by metformin and aspirin, especially when they are used in combination.

Recently, high-throughput RNA sequencing (RNA-seq) is rapidly becoming an attractive alternative to hybridization-based microarrays for transcriptome profiling. This approach enables investigators to get a relatively unbiased and precise measurement of levels of transcripts and their isoforms than other methods^13^. One of the main goals of RNA-seq is to identify the differentially expressed genes in two or more conditions. Such genes are selected based on a combination of expression change threshold and score cutoff, which are usually based on P values generated by statistical modeling.

In order to depict a comprehensive picture of the mechanisms underlying the antineoplastic activity of metformin and aspirin, it is necessary to obtain some new insights, on a whole-transcriptome level, on the gene regulation by metformin and aspirin. In this study, using RNA-seq technology, we comprehensively analyzed the differential gene expression induced by metformin (5 mM) and aspirin (2 mM), alone or in combination, after treatment of pancreatic cancer cell line PANC-1 cells for 48 hours. We identified the top genes and the most related biological networks and pathways which are not or seldom reported previously, to be modulated by metformin and aspirin. Future function validation is warranted to verify our results *in vitro* and *in vivo*.

## Results

### Overview and validation of differentially expressed genes induced by metformin and aspirin

**Figure 1:**
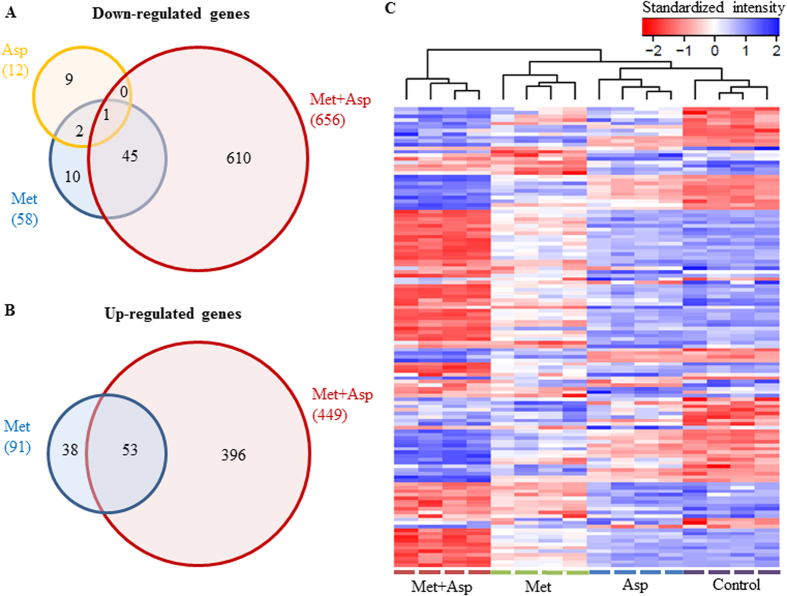
Overview of genes regulated by metformin and aspirin. (**A**,**B**) Venn diagrams comparing the down-regulated (**A**) and up-regulated (**B**) genes after treatments of metformin and aspirin, alone or the combination. (**C**) The clustering heat map of 16 samples based on the 150 top differentially expressed genes across 4 groups (untreated control, metformin, aspirin and metformin plus aspirin). Each column is labeled with different colors according to the sample type. Met, metformin; Asp, aspirin

**Figure 2:**
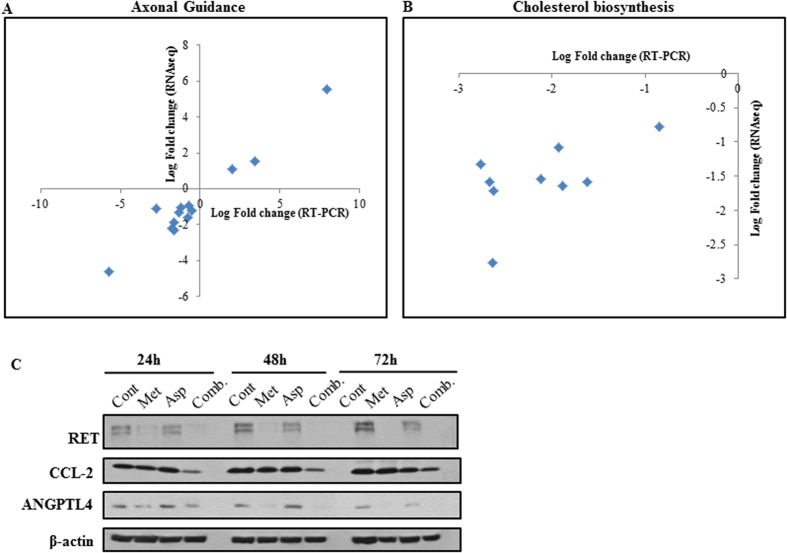
Validation of the mRNA and protein changes of the selected genes. Nine genes in the “Superpathway of Cholesterol Biosynthesis” (**A**) and thirteen genes in the “Axonal Guidance Signaling” (**B**) were randomly selected and their mRNA levels were determined by qRT-PCR in PANC-1 cells untreated or treated with the combination of metformin (5 mM) and Aspirin (2 mM) for 48 h. The protein levels of the selected genes RET, CCL2, and ANGPTL4 were determined by Western blotting (**C**). PANC-1 cells were treated with metformin (5 mM) and aspirin (2 mM), alone or in combination for 24, 48 and 72 h, respectively. β-actin was used as a loading control. Cont, control; Met, metformin; Asp, aspirin; Comb, the combination of metformin and aspirin.

To determine the impact of metformin and aspirin on the whole transcriptome, we compared the global gene expression profiles of untreated PANC-1 cells to those treated by metformin, aspirin, or a combination. We detected and quantified 11,551 unique genes, and compared the gene expression profiles of all three drug-treated samples to the untreated sample. Differentially expressed genes were identified by arbitrarily using a 2.0-fold-change cut-off value (*P* value < 1 × 10^−5^) compared to the untreated PANC-1 cells. We identified 149 differentially expressed genes (58 down-regulated and 91 up-regulated) from the metformin treated cells and 12 genes (all down-regulated) from the aspirin treated cells. Moreover, we identified 1,105 genes (656 down-regulated and 449 up-regulated) that have significantly different expression levels in the metformin-aspirin-combination treated cells. Of the genes downregulated by metformin alone, 79.3% (46/58) were also down-regulated by the combination of metformin and aspirin (Fig. 1A), while 58.2% (53/91) of the up-regulated genes by metformin also appeared in the subset of up-regulated genes by the combination (Fig. 1B). However, the genes regulated by aspirin alone did not show much commonality with those by metformin alone or the combination of metformin and aspirin. Only 25.0% (3/12) and 8.3% (1/12) of the down-regulated genes by aspirin were also down-regulated in metformin treated cells and metformin-aspirin treated cells, respectively (Fig. 1A). As shown in Fig. 2C, the clustering of top differentially expressed genes showed clearly distinguishable patterns across four conditions.

To validate the results from the RNA sequencing data, we randomly selected 10 genes found to be regulated by the combination of metformin and aspirin to determine their mRNA levels by using quantitative reverse transcription-PCR (qRT-PCR) (Supplementary Table S1). Our data showed that the results from qRT-PCR were consistent with those from the RNA-seq for all 10 selected genes, confirming that comparative RNA-seq analysis is a quantitative approach.

### Top differentially expressed genes regulated by metformin and aspirin

Using a 2.0-fold-change cut-off value (P value < 10^−5^) relative to the transcriptome of the untreated PANC-1 cells, we identified genes that showed significant expression changes upon the treatment of metformin, aspirin or both. The top 10 down-regulated or up-regulated genes by metformin, aspirin or both were shown in Table 1 and Table 2, respectively. The full data set is available at the National Centre for Biotechnology Information Gene Expression Omnibus database (www.ncbi.nlm.nih.gov/geo/, accession number: SRP056109). Among the top down-regulated genes, *ANGPTL4, FAM222A, RET* were down-regulated to more than 4 folds upon metformin treatment (Table 1). *DNAH10OS, LAMP3, PLIN4, DUSP15, HMOX1* are the top five down-regulated genes upon aspirin treatment (Table 1). *PCDH18, CCL2, RASL11A, FAM111B, BMP5* were down-regulated to more than 20 folds by the combination of metformin and aspirin (Table 1). *RET, CDH18* and *PCDH18* down-regulated by metformin were also found to be down-regulated by the combination of metformin and aspirin (Table 1). Of the genes up-regulated by metformin alone, *GRB7, GUCA1B, SGK2, PPP1R32* were up-regulated to more than 4 folds compared to the untreated control (Table 2). *NGFR, NPTX1, C7orf57, MRPL23AS1*, and *UNC5B* were up-regulated to more than 10 folds by the combination of metformin and aspirin (Table 2). However, we did not detect any genes that were significantly up-regulated by aspirin alone.

### The main gene networks and canonical pathways modulated by metformin and aspirin

To investigate the possible biological function and pathways that were regulated by metformin and aspirin, we employed Ingenuity Pathway Analysis (IPA) software to do the “core analysis” for all the genes significantly up-regulated or down-regulated by metformin, aspirin or the combination. We first analyzed the gene networks that were most regulated by metformin, aspirin or the combination, respectively.

The top two gene networks that were regulated by metformin were: a) “Cardiovascular System Development and Function, Cellular Movement, Cellular Assembly and Organization” (score = 40); and b) “Cardiovascular System Development and Function, Cellular Movement, Skeletal and Muscular System Development and Function” (score = 38) (Supplementary Fig. S1). The first gene network was identified around *Ras* subfamily, *TGF-β, FAK* and *ERK1. Ras* has been found to be mutated in up to 90% of pancreatic cancers, and ERK1is part of Ras-ERK signaling pathway which is critical for pancreatic carcinogenesis. The second network was around PI3-K/Akt, interferon-α, pro-inflammatory cytokines, AMPK and Notch. PI3-K/Akt is an important tumor cell survival pathway, and interferon-α exhibits inhibitory effects on tumor cell growth.

The top gene network that was regulated by aspirin was “Cellular Compromise, Lipid Metabolism, Small Molecule Biochemistry” (score = 27) (Supplementary Fig. S2). The genes in this network were around *TNF, HMOX1* and three perilipin family members, *PLIN2, PLIN3* and *PLIN4*. TNF has been reported to modulate pancreatic cancer cell growth by affecting tumor-infiltrating macrophages, and HMOX1 protects against oxidative stress and modulates inflammation and angiogenesis.

The top two gene networks that were regulated by the combination of metformin and aspirin were: a) “Cell Cycle, Cell Morphology, Cellular Assembly and Organization” (score = 40), and b) “Cellular Assembly and Organization, Drug Metabolism, Small Molecule Biochemistry” (score = 35) (Supplementary Fig. S3). The first gene network was identified around *Histone h3*, which is involved in the structure of chromatin in eukaryotic cells, and *RBBP4*, which binds to histones and participates in histone acetylation and chromatin assembly. The second one was identified around *VEGF*, a main angiogenesis regulator that has been targeted for cancer therapy for years.

In addition, we used the IPA software to determine the canonical pathways modulated by the combination of metformin and aspirin (Table 3). “Superpathway of Cholesterol Biosynthesis”, “Cell Cycle: G1/S Checkpoint Regulation”, and “Axonal Guidance Signaling” are the top three most statistically significant canonical pathways regulated by the combination of metformin and aspirin. We randomly selected 9 genes in the “Superpathway of Cholesterol Biosynthesis” and 13 genes in the “Axonal Guidance Signaling” and determined their mRNA levels in PANC-1 cells untreated or treated by the combination of metformin and aspirin by qRT-PCR (Fig. 2A,B). The results further confirmed the down-regulation or up-regulation of these genes upon the treatment of metformin and aspirin. Furthermore, we selected to detect the protein levels of several genes which showed significant changes in the mRNA levels (Fig. 2C). The results showed that the protein levels of ANGPTL4 and RET decreased along with the downregulation of mRNA of these two genes. We only observed the down-regulation of CCL2 in the cells treated by the combination, which is also in accordance with the RNA-seq results.

## Discussion

In this study, we established, for the first time, a global transcriptome profile related to metformin, aspirin, and the combination of both in pancreatic cancer cells. We found that while metformin or aspirin alone only slightly changed the transcriptome profile of PANC-1 cells (149 and 12 genes, respectively), the combination of metformin and aspirin dramatically affected the transcription of 1,105 genes. We recently demonstrated that the combination of metformin and aspirin, at relatively low concentrations, significantly inhibited the cell viability in pancreatic cancer cell lines PANC-1 and BxPC3^14^. These results indicate that the combination of metformin and aspirin exerts synergistic action on the transcriptome of PANC-1 cells and thus affects their proliferation and survival. It will be interesting to further examine the synergistic effects of the combination and the underlying mechanisms in the future.

To determine the optimum concentrations of metformin and aspirin used in this study, we first accessed the cell viability of pancreatic cancer cells upon the treatment of metformin and aspirin^14^. The combination of metformin and aspirin caused a synergistic inhibition of cell viability in both PANC-1 and BxPC-3 cells, mainly in low dosages of both drugs [e.g., PANC-1 cells: 5 mM metformin and 2 mM aspirin, and 10 mM metformin and 4 mM aspirin (P for the test of synergy = 0.034 and 0.007, respectively); BxPC-3 cells: 1 mM metformin and 0.25 mM aspirin, and 5 mM metformin and 0.5 mM aspirin (P for the test of synergy = 0.004 and 0.045, respectively)]. On the other hand, to our best knowledge, the plasma salicylate (the *in vivo* metabolite of aspirin) concentration in humans is 0.5–2.5 mM^10^,^15^, and the plasma concentration of metformin is about 16 μM^16^. Considering the results of cell viability and physiological concentration of metformin and aspirin that can be achieved *in vivo*, we chose 5 mM metformin and 2 mM aspirin as the concentration used in this study. Although the dose of metformin we had used is still higher that the physiologically achievable plasma concentration, metformin has been reported to accumulate in tissues at concentrations several folds higher than those in blood^17^,^18^. In addition, concentration of metformin in the mitochondrial matrix could achieve a concentration higher than 20 mM^18^ due to the positive charge of metformin. Taken together, it suggested that the concentrations we used in *in vitro* models might be attained during cancer treatment.

Of the top down-regulated genes in metformin treated cells, *ANGPTL4* (angiopoietin-like 4) is the most dramatically down-regulated genes. ANGPTL4 has been reported as a tumor suppressor through inhibiting angiogenesis in gastric cancer^19^. Down-regulation of *ANGPTL4* mRNA in hepatocellular carcinoma has been shown to be associated with advanced tumor stage, tumor recurrence, and poor postoperative^20^. Interestingly, metformin treatment was shown to attenuate the increased mRNA levels of *ANGPTL4* in type I diabetic mice^21^. However, the precise function and role of ANGPTL4 in pancreatic cancer is still unclear.

It is worth noting that RET was dramatically down-regulated in both metformin treated cells (5 fold down-regulation *vs.* untreated cells) and metformin-aspirin-combination treated cells (14 fold down-regulation *vs.* untreated cells). RET, a single-pass transmembrane receptor tyrosine kinase (RTK), is transcriptionally regulated by DNA-binding factors that modulate basal transcription, such as SP1, SP3 and early growth response protein 1 (EGR1)^22^. Metformin was reported to decrease SP1/SP3 expression and their downstream targets, such as Bcl-2, survivin, and cyclin D1, in pancreatic cancer cells^23^. Recently, Nair *et al.* reported that the regulation of Sp transcription factors could be a novel mechanism by which metformin inhibits IGF-1R/mTOR and EGFR/K-Ras signaling^24^. Thus, it is possible that metformin regulates the transcription of RET through SP transcription factors. RET is expressed in 50–65% of pancreatic ductal carcinomas and is correlated to an advanced metastatic status^22^. Increased RET activity stimulates cell proliferation and transformation by promoting tumor-associated inflammation and recruiting primary immune cells to the tumor microenvironment^22^. Interestingly, STAT3 which has been shown to play an important role in pancreatic tumorigenesis, can also be activated by RET^25^. Metformin has been shown to inhibit STAT3 activity in various cancers^2^. However, the underlying mechanism by which metformin inhibits STAT3 is still unclear. Although further function validation studies are needed, the inhibition of RET by metformin provides a new possible explanation for the molecular mechanisms of the inhibition of STAT3 induced by metformin.

Additionally, we observed that CCL2 was dramatically down-regulated (50 fold down-regulation *vs.* untreated cells) in metformin-aspirin-combination treated cells. CCL2, an inflammatory chemokine, is closely connected with tumor associated macrophage (TAM) infiltration and cancer progression. CCL2 is overexpressed in various cancer types such as lung, breast and prostate cancer^26^. In pancreatic cancer, high CCL2 expression is associated with significantly decreased survival^27^. CCL2 secreted by tumor cells can recruit monocytes and TAMs. These tumor-infiltrating inflammatory cells form a tumor-protective microenvironment, thus enhancing tumor growth and metastasis^26^,^28^,^29^. Therefore, inhibition of CCL2 might be a new therapeutic approach for the prevention and treatment of pancreatic cancer. Given that both metformin and aspirin are used in combination with other chemotherapeutic drugs in ongoing clinical trials, it is of interest to explore the possibility of the use of metformin and aspirin as adjuvant cancer immunotherapy.

Of the genes that most dramatically up-regulated in metformin treated cells, *SGK2* (serum/glucocorticoid regulated kinase 2) was reported to be important for cell proliferation and viability of HPV-positive cervical cancer cell lines^30^. *GRB7* (growth factor receptor-bound protein 7) was found to be overexpressed in pancreatic tumor than normal pancreatic tissue and was associated with regional lymph node metastatic spread of pancreatic cancer^31^. However, the modulation of these two genes by metformin has not been previously investigated. WISP2 (WNT1 inducible signaling pathway protein 2) has been reported to be up-regulated in metformin-adapted MCF-7 cells^32^, which is consistent with our finding. Loss of WISP2 expression was associated with pancreatic cancer progression as WISP2 might prevent against epithelial to mesenchymal transition^33^. Further studies are warranted to investigate the role of metformin-induced WISP2 up-regulation in pancreatic cancer prevention and treatment.

Of the genes that most dramatically up-regulated in metformin-aspirin-combination treated cells, none of them has been previously reported to be regulated by metformin or aspirin. NGFR (nerve growth factor receptor) exerts a tumor-suppressing effect in bladder, stomach, liver, colorectal and prostate cancers while it promotes tumor progression in brain tumors and melanomas^34^. It has been reported that NGFR was overexpressed in pancreatic tumor compared to normal pancreatic tissue^35^,^36^. However, the exact role of NGFR in pancreatic cancer development is still unclear. NPTX1 (neuronal pentraxin I) has been shown to be silenced through methylation in 5′ CPG islands in pancreatic cancer cell lines^37^. Similar results were also obtained in colorectal tumors^38^. UNC5B has been identified as a tumor suppressor, which can induce apoptosis through p53-dependent manner in various cancer cells^39^. Reduced UNC5B expression is correlated with higher recurrence rate and poorer prognosis in both bladder and colorectal cancers^40^,^41^.

We observed only 12 genes were down-regulated, and none of the genes was up-regulated by aspirin. These minimal effects of aspirin on changes in gene expression might be due to the relatively low concentration which we used in the experiments. On the other hand, it is possible that the effects of aspirin on gene expression may be due to the post-translational modification of proteins, such as acetylation. It has been shown that aspirin causes the inactivation of cyclooxygenases (COX) through the acetylation on serine residues^42^,^43^. Aspirin could also acetylate p53 and induce the expression of p21 and Bax in breast cancer cells^44^. Interestingly, aspirin also acetylated multiple cellular proteins in colon cancer cells including histone H1b and histone H4^45^, suggesting that aspirin may participate in the regulation of gene expression by modulating histone acetylation. In fact, aspirin was reported to induce the HDAC Sirtuin 1 through the production of hydrogen peroxide^46^, and promote the transcription of netrin-1 by enhancing histone acetylation^47^. These results suggest that the aspirin-induced changes of gene expression might be partially due to the shifting of equilibrium of the acetylation/deacetylation process. Additionally, aspirin is readily broken down in the body to salicylic acid, the main metabolite of aspirin. Mohammed *et al.* showed that salicylic acid induces greater caspase activity than aspirin in HT3 cervical cancer cell line^48^. It is possible that salicylic acid may have stronger effects than aspirin on modulation of gene expression. Further studies are warranted to compare the effects of aspirin and salicylic acid on the gene expression pattern in pancreatic cancer cells.

The canonical pathway analysis highlighted “Superpathway of Cholesterol Biosynthesis” is the most significantly regulated pathway in PANC-1 cells treated by the combination of metformin and aspirin. Cholesterol is a crucial component of cell membranes and the cholesterol levels are relatively high in cancer cells due to the needs of their high proliferation rate^49^,^50^. Also, cholesterol is essential for the assembly of lipid rafts, which regulate various signaling pathways intimately related to carcinogenesis and metastasis, such as Fas receptor, TRAIL, Akt and CD44^51^. In our study, 11 of the 28 molecules in “Superpathway of Cholesterol Biosynthesis” were down-regulated while other molecules did not show significant change. Of the 11 molecules, MVK, PMVK and MVD are involved in the formation of isopentenyl- pyrophosphate, which is the substrate of cholesterol synthesis; CYP51A1, DHCR7, DHCR24 and EBP are enzymes that catalyze the formation of cholesterol from lanosterol, which is the terminal process of cholesterol biosynthesis. The down-regulation of these genes indicates a possible reduction of the overall cholesterol synthesis in tumor cells. Metformin has been reported to inhibit fatty acid synthesis by decreasing the expression of acetyl CoA carboxylase, fatty acid synthase and citrate lyase, which is related to the blocking of tumorigenesis^52^,^53^. Recently, metformin was also shown to inhibit the cholesterol biosynthesis rate in macrophages^54^. Our results further support the inhibitive effect by metformin on cholesterol biosynthesis and provide more potential molecular targets of metformin in this pathway. Considering the importance of cholesterol synthesis in tumor development, the remodeling of the cholesterol metabolism by metformin and aspirin may be a novel mechanism of their antitumor activity.

Another canonical pathway worth noting is “Axonal Guidance Signaling”. Axonal guidance pathway plays an important role in neuronal extension and location during embryo development^55^. Recently, accumulating evidence indicates that axonal guidance pathway is also involved in tumor development and progression by regulating tumor cell migration, cell death and angiogenesis in various cancers, including pancreatic cancer^56^. UNC5A, UNC5B and UNC5C, which are the receptors of netrin 1, are all down-regulated in various cancers through promoter methylation^56^. However, the mechanism by which UNC5 proteins exert tumor suppressive effects is still unclear. According to our results, the expression of UNC5B in metformin-aspirin treated cells is up-regulated to 11 folds compared to untreated cells, indicating a possible role of UNC5B in the anti-cancer effect induced by the combination of metformin and aspirin. We also showed that four members of the class 3 semaphorin family were down-regulated by metformin and aspirin, including two pro-tumor semaphorins, SEMA3C and SEMA3E. Both SEMA3C and SEMA3E have been shown to be overexpressed in various cancers^57^. The overexpression of SEMA3C and SEMA3E in cancer cells correlates with multidrug resistance and increased metastatic capability^58^. Since axonal guidance pathway involves hundreds of genes, further investigations are needed to elucidate the effect of metformin and aspirin on this signaling pathway.

In summary, our study provided a quantitative gene expression profile of a pancreatic cancer cell line treated with metformin and aspirin, alone or in combination. We also analyzed the altered biological networks and pathways induced by metformin and aspirin. These findings provide new insight for the exploration of molecular mechanisms underlying the anti-cancer effect of metformin and aspirin in pancreatic cancer. However, future studies are needed to verify whether these altered gene expressions occur under physiologic conditions and contribute to the antineoplastic activity of metformin and aspirin in pancreatic cancer or other cancers.

## Materials and Methods

### Cell culture

Human pancreatic cancer cell line PANC-1 was purchased from the American Type Culture Collection (Manassas, VA, USA). PANC-1 cells were maintained in Dulbecco’s Modified Eagle’s Media (DMEM) (Sigma-Aldrich, St Louis, MO, USA) supplemented with 10% heat-inactivated fetal bovine serum (FBS) (Sigma-Aldrich, St Louis, MO) at 37 °C and 5% CO_2_. PANC-1 cells were seeded in 6-well plates (5 × 10^5^ cells per well). 24 hours later, cells were untreated or treated by 5 mM metformin, 2 mM aspirin or a combination of metformin (5 mM) and aspirin (2 mM) for 48 hours.

### mRNA library preparation and sequencing

Total RNA was isolated from untreated and treated PANC-1 cells with TRIzol reagent (Invitrogen, Carlsbad, CA, USA) followed by clean up with RNeasy Mini kit (Qiagen, Valencia, CA, USA), according to the manufacturer’s instructions. Quantity and quality of DNase treated RNA were determined using Agilent 2100 Bioanalyzer (RNA Nano 6000) and Nanodrop, respectively. A total of 16 RNA samples were sequenced [4 samples per condition × 4 conditions (untreated control, metformin, aspirin or a combination of metformin and aspirin)] using 500 ng of total RNA as input. Library preparation and RNA sequencing was carried out using the Illumina TruSeq™ RNA Sample Prep Kit v2 according to the manufacturer’s protocol (Illumina, San Diego, CA, USA). Samples were sequenced on the Illumina HiSeq 2500, 2 × 100 bp paired-end reads, to a minimum depth of 30 million reads per sample.

### Computational analyses of RNA-seq data

A total of 240 million obtained reads of high quality clean tags were mapped and annotated human reference genome using Bioconductor package *biomaRt* (http://www.bioconductor.org)^59^. Mapped reads with mapping quality 10 or more were defined as uniquely mapped reads and used in the downstream analyses. Averagely, about 70.0% reads can be uniquely mapped to the annotated human genome. Ranking of genes by degree of differential expression was performed using the Bioconductor edgeR package version 2.14 (http://www.bioconductor.org) and in-house code developed in the R statistical language (http://www.r-project.org). Significant genes were arbitrarily identified by using the cutoff values of *P *≤ 1 × 10^−5^ and fold change (FC) in mean expression of FC ≥ |2|.

### Biological network and pathway analysis

Biological networks and pathways related to metformin, aspirin and the combination were analyzed with Ingenuity Pathway Analysis (IPA) software (Qiagen, CA, USA). The lists of all genes identified in gene expression analysis were uploaded into the IPA software. For the analysis of networks and pathways, the cutoff values are set as *P* ≤ 1 × 10^−5^ and FC ≥ |2|.

### Validation of RNA-seq results by qRT-PCR

Quantitative RT-PCR was used to validate the differentially expressed genes. Expression of mRNA was determined in all 4 samples using Power SYBR® Green RNA-to-CT™ 1-Step Kit (Life Technologies, CA, USA) according to the manufacturer’s protocol. All of the primers used in qRT-PCR were purchased from Qiagen (Limburg, Netherland), and reference numbers used are: NPTX1 (PPH10301B), SLC20A1 (PPH11164A), DUSP15 (PPH18348B), HES7 (PPH20383B), NGFR (PPH00821A), PCDH18 (PPH10623A), CCL2 (PPH00192F), RASL11A (PPH14046A), FAM111B (PPH13923A), BMP5 (PPH00509A), GAPDH (PPH00150F), ACAT2 (PPH01591B), CYP51A1 (PPH01246A), DHCR7 (PPH06336F), EBP (PPH14187B), PMVK (PPH06350B), MVK (PPH06339A), DHCR24 (PPH02278F), FDPS (PPH01995A), LSS (PPH06366A), TM7SF2 (PPH08913B), LINGO1 (PPH08561A), EPHA7 (PPH05721F), FZD4 (PPH02427B), WNT7B (PPH02464C), PIK3R3 (PPH02312A), EFNB2 (PPH01144A), ROBO1 (PPH05962A), NRP1 (PPH01152A), PRKCA (PPH00977C), ABLIM3 (PPH15859A).

### Western Blotting

The Western blotting for the selected proteins was performed, as previously described^14^. Briefly, the treated and untreated cells were rinsed with PBS and extracted on ice with cell lysis buffer (Cell signaling, Beverly, MA, USA). The protein concentrations were determined with BCA Protein Assay Kit (Thermo scientific, Waltham, MA, USA). 20 μg of total proteins from each sample were loaded and separated on a gradient 4–20% polyacrylamide gel and transferred to polyvinylidene difluoride (PVDF) membrane. Membranes were blocked with 5% fat-free milk in Tris-buffered saline-Tween 20 and incubated with the primary antibodies against RET and CCL2 from Cell Signaling Technology (Beverly, MA, USA), and antibody against ANGPTL4 from Santa Cruz Biotechnology (Santa Cruz, CA, USA). Blots were subsequently washed and incubated with the appropriate HRP-conjugated secondary antibodies. The immunoreactive bands were visualized by enhanced chemiluminescence (Thermo Fisher, Rockford, IL USA) according to the manufacturer’s instructions. The levels of β-actin were estimated to check for equal samples loading.

### Statistical Analysis

The results of RT-PCR were normalized to expression of GAPDH using the formula 2^∆ CT^. One-way ANOVA was used for comparing treatment with the combination of metformin and aspirin to the untreated control. A *P* value less than 0.05 was considered statistically significant.

## Additional Information

**How to cite this article**: Yue, W. *et al.* Transcriptomic analysis of pancreatic cancer cells in response to metformin and aspirin: an implication of synergy. *Sci. Rep.* **5**, 13390; doi: 10.1038/srep13390 (2015).

## Supplementary Material

Supplementary Information

## Figures and Tables

**Table 1:** Top 10 down-regulated genes in PANC-1 cells treated by metformin and aspirin.

Gene ID	Fold Change	P-value	Function
*Metformin*			
ANGPTL4	0.14	2.95E-25	glucose homeostasis, lipid metabolism, insulin sensitivity, vascular growth, tumor cell invasion
FAM222A	0.18	2.34E-19	unknown
RET	0.21	1.76E-22	signaling transduction, cell growth and differentiation
SCDP1	0.31	1.76E-07	pseudogene
CDH18	0.32	6.01E-11	calcium-dependent cell-cell adhesion, synaptic adhesion, axon outgrowth and guidance
RCBTB2	0.33	3.04E-11	guanine nucleotide exchange
FADS2	0.33	1.12E-42	unsaturation of fatty acids
PCDH18	0.34	3.55E-13	cell-cell connections in brains
FOXQ1	0.35	4.03E-07	regulates epithelial-mesenchymal transition
NEU1	0.35	7.76E-17	lysosomal enzyme
*Aspirin*			
DNAH10OS	0.33	1.22E-06	unknown
LAMP3	0.35	4.58E-10	dendritic cell function and adaptive immunity
PLIN4	0.36	1.50E-10	lipid distribution and metabolism
DUSP15	0.40	2.39E-06	oligodendrocyte differentiation
HMOX1	0.40	1.81E-07	heme catabolism
FTLP3	0.41	1.90E-06	pseudogene
PLIN2	0.42	2.06E-18	lipid distribution and metabolism
APOC1	0.44	5.00E-11	cholesterol metabolism, membrane remodeling, neuronal apoptosis and reorganization
SDSL	0.46	9.25E-07	low serine dehydratase and threonine dehydratase activity
SLC44A2	0.46	8.94E-18	some choline transporter activity
*Combination*			
PCDH18	0.01	1.03E-64	cell-cell connections in brains
CCL2	0.02	3.96E-166	macrophage infiltration
RASL11A	0.03	9.34E-58	Regulator of rDNA transcription
FAM111B	0.04	7.69E-42	unknown
BMP5	0.04	8.27E-35	dendritic growth
SKIDA1	0.05	4.55E-39	unknown
CDH18	0.07	3.10E-29	calcium-dependent cell-cell adhesion, synaptic adhesion, axon outgrowth and guidance
AGR2	0.07	1.17E-26	regulating cell proliferation, promoting cell growth
RET	0.07	5.93E-49	signaling transduction, cell growth and differentiation
RXFP4	0.08	4.66E-23	high affinity receptor for INSL5, inhibit cAMP accumulation

**Table 2:** Top 10 up-regulated genes in PANC-1 cells treated by metformin and aspirin.

Gene ID	Fold Change	P-value	Function
*Metformin*			
GRB7	6.48	1.41E-11	integrin signaling pathway, cell migration
GUCA1B	5.49	2.86E-18	activates photoreceptor guanylate cyclases
SGK2	4.48	4.41E-12	membrane transporters, cell growth, survival and proliferation
PPP1R32	4.19	7.49E-10	unknown
WISP2	3.33	1.11E-11	Downstream of WNT1 signaling pathway
JAK3	3.18	4.17E-11	Stat3 pathway
TCP11L2	3.01	3.54E-11	unknown
AMT	3.00	4.49E-18	glycine cleavage
NT5M	2.93	7.16E-10	Dephosphorylation
HES7	2.93	7.44E-09	transcriptional repressor
*Combination*			
NGFR	47.48	1.69E-122	cell survival and differentiation
NPTX1	20.17	1.80E-46	activates photoreceptor guanylate cyclases
C7orf57	14.50	8.02E-41	unknown
MRPL23AS1	12.39	4.49E-36	unknown
UNC5B	11.20	2.37E-51	guidance of nerves/blood vessels, induce apoptosis
DUSP15	8.91	2.66E-60	oligodendrocyte differentiation
HES7	8.30	1.80E-33	transcriptional repressor
MTURN	7.74	8.44E-24	early neuronal development
SLC20A1	6.72	9.85E-36	cellular metabolism, signal transduction, and nucleic acid and lipid synthesis, extracellular matrix and cartilage calcification as well as in vascular calcification
GPCPD1	6.42	6.24E-49	unknown

**Table 3:** Top 5 canonical pathways of the genes regulated by metformin and aspirin.

Ingenuity Canonical Pathways	Ratio	P-value	Target genes
Superpathway of Cholesterol Biosynthesis	11/28 (0.393)	2.75E-08	MVD,FDPS,EBP,DHCR7,PMVK,ACAT2,DHCR24,MVK,LSS,TM7SF2,CYP51A1
Cell Cycle: G1/S Checkpoint Regulation	11/64 (0.172)	2.00E-04	CCND2*,TFDP1,**HDAC11**,SUV39H1,E2F1,TGFB3,TGFB2,CDKN2C,E2F2,**HDAC5**,CDC25A
Axonal Guidance Signaling	37/433 (0.085)	4.57E-04	**FYN**,TUBA1B,**ADAM17**,MYL6,**PDGFA**,PIK3R1,**UNC5B**,PLCH2,**VEGFA**,EFNB2,GNG11,**NGFR**,WNT7B,**ABLIM3**,PRKAR1B,TUBA1C,PLXNB3,SEMA3F,UNC5C,**PRKCA**,BMP1,EPHA7,SEMA3E,TUBB4B,PLXND1,BMP5,PIK3R3,SEMA3A,TUBA1A,FZD4,TUBB6,LINGO1,NFATC2,**PIK3CD**,SEMA3C,**BMP6**,NRP1
Chronic Myeloid Leukemia Signaling	13/93 (0.140)	4.57E-04	PIK3R3,**GAB2**,TFDP1,**HDAC11**,SUV39H1,PIK3R1,E2F1,TGFB2,TGFB3,**PIK3CD**,IKBKE,E2F2,**HDAC5**
Pancreatic Adenocarcinoma Signaling	14/106 (0.132)	5.01E-04	PIK3R3,**VEGFA**,**HMOX1**,TFDP1,SUV39H1,PIK3R1,E2F1,TGFB2,TGFB3,**PIK3CD**,**PLD6**,**JAK2**,**JAK3**,E2F2

^*^Up-regulated genes are in bold type.
