# Supplementary material for: Transcriptomic analysis of pancreatic cancer cells in response to metformin and aspirin: an implication of synergy
Source: Sci Rep. 2015 Aug 21;5:13390. doi: 10.1038/srep13390 (PMC4543968; doi:10.1038/srep13390)
Supplement: Supplementary Information [file srep13390-s1.pdf]

Supplementary Information

Title: Transcriptomic analysis of pancreatic cancer cells in response to metformin and aspirin: an implication of synergy

Authors: Wen Yue[1], Tao Wang[2], Emmanuel Zachariah[1], Yong Lin[1,3], Chung S. Yang[1,4], Qing Xu[5], Robert S. DiPaola[1], Xiang-Lin Tan[1, 6, †]

Supplementary Table S1. Validation of differentially expressed genes in untreated cells and metformin-aspirin treated cells.

Gene ID	RNA sequencing		qRT-PCR	
	Fold change	P value	Fold change	P value*
CCL2	0.02	3.96E-166	0.03	< 0.0001
RASL11A	0.03	9.34E-58	0.04	< 0.0001
CDH18	0.07	3.10E-29	0.07	< 0.001
AGR2	0.07	1.17E-26	0.03	< 0.0001
CDH3	0.45	1.74E-6	0.56	< 0.05
CDH1	3.38	1.86E-17	4.81	< 0.001
GPR56	6.26	3.68E-39	13.50	< 0.0001
GPCPD1	6.42	6.24E-49	7.26	< 0.0001
HES7	8.30	1.80E-33	2.35	< 0.001
NPTX1	20.17	1.80E-46	6.41	< 0.0001

* Using one-way ANOVA for comparing treatment with the combination of metformin and aspirin to the untreated control.

Cardiovascular System Development and Function, Cellular Movement, Cellular Assembly and Organization	Cardiovascular System Development and Function, Cellular Movement, Skeletal and Muscular System Development and Function
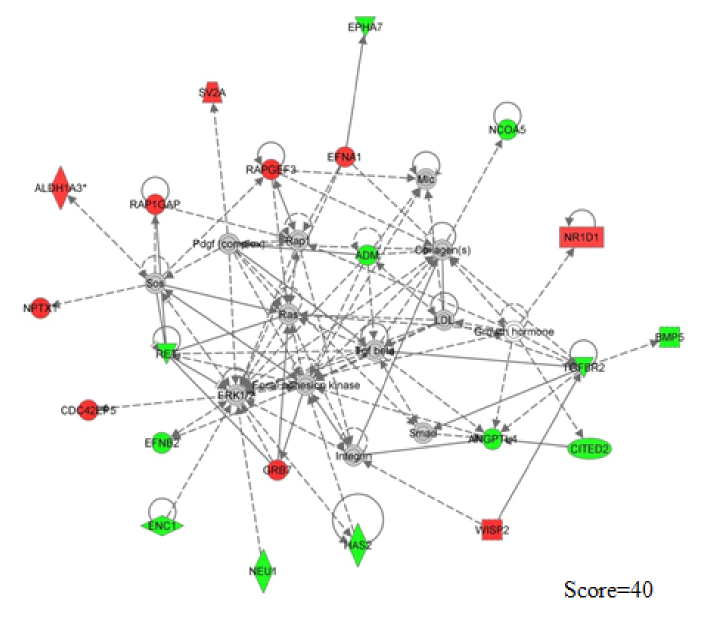	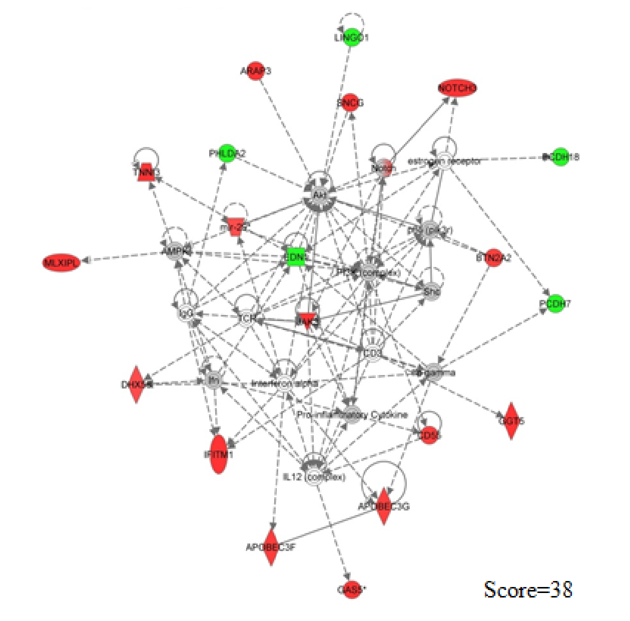

Supplementary Figure S1. Network analysis of genes regulated by metformin using IPA software. The networks with the highest 2 scores (which indicates the statistically significance of the interconnection between the molecules depicted in the network) were shown. The color of the molecules indicates the gene expression (red: upregulation; green: downregulation). Continuous lines represent direct interactions while dashed lines represent indirect interactions.

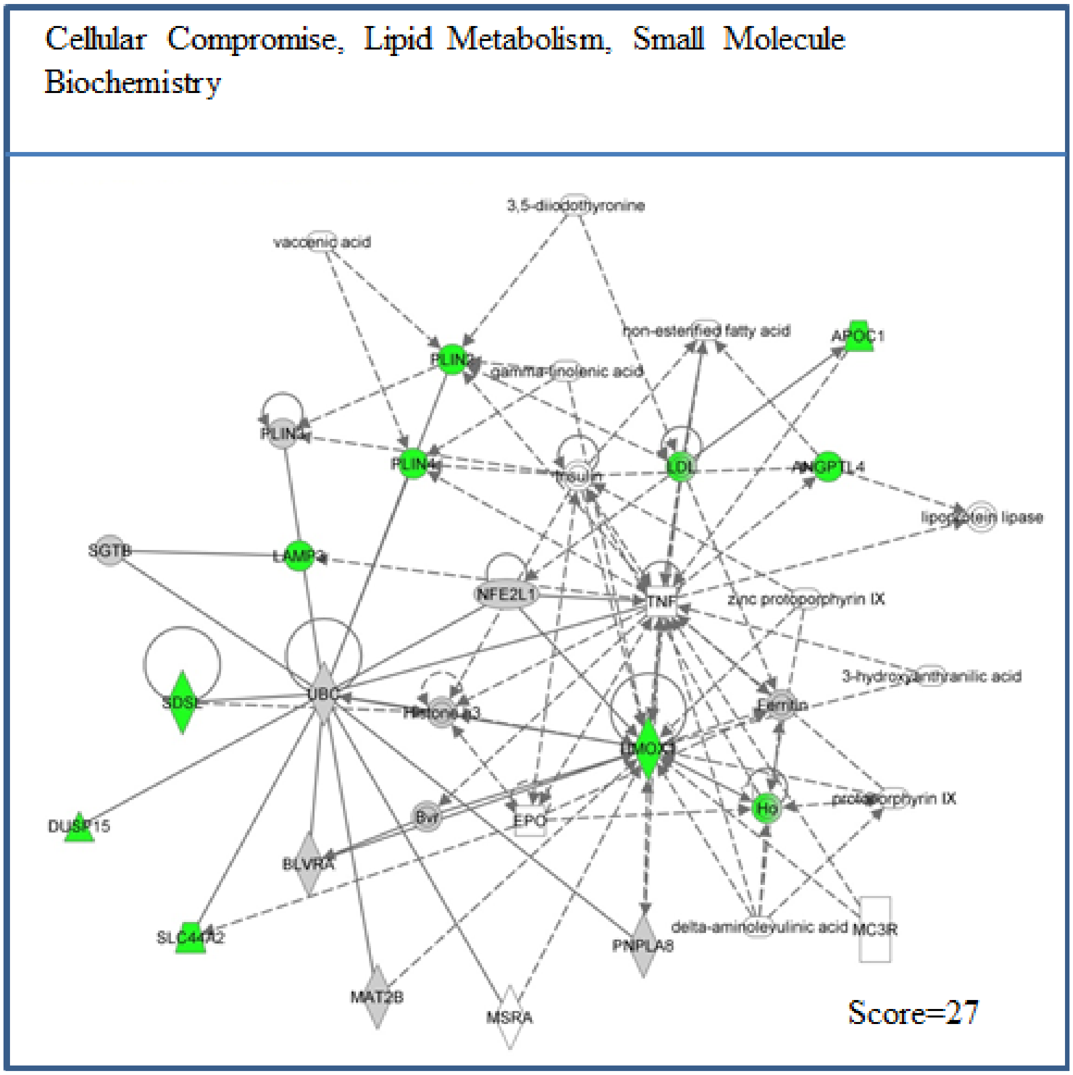

Supplementary Figure S2. Network analysis of genes down-regulated by aspirin using IPA software. The network with the highest scores (which indicates the statistically significance of the interconnection between the molecules depicted in the network) were shown. The color of the molecules indicates the gene expression (green: downregulation). Continuous lines represent direct interactions while dashed lines represent indirect interactions.

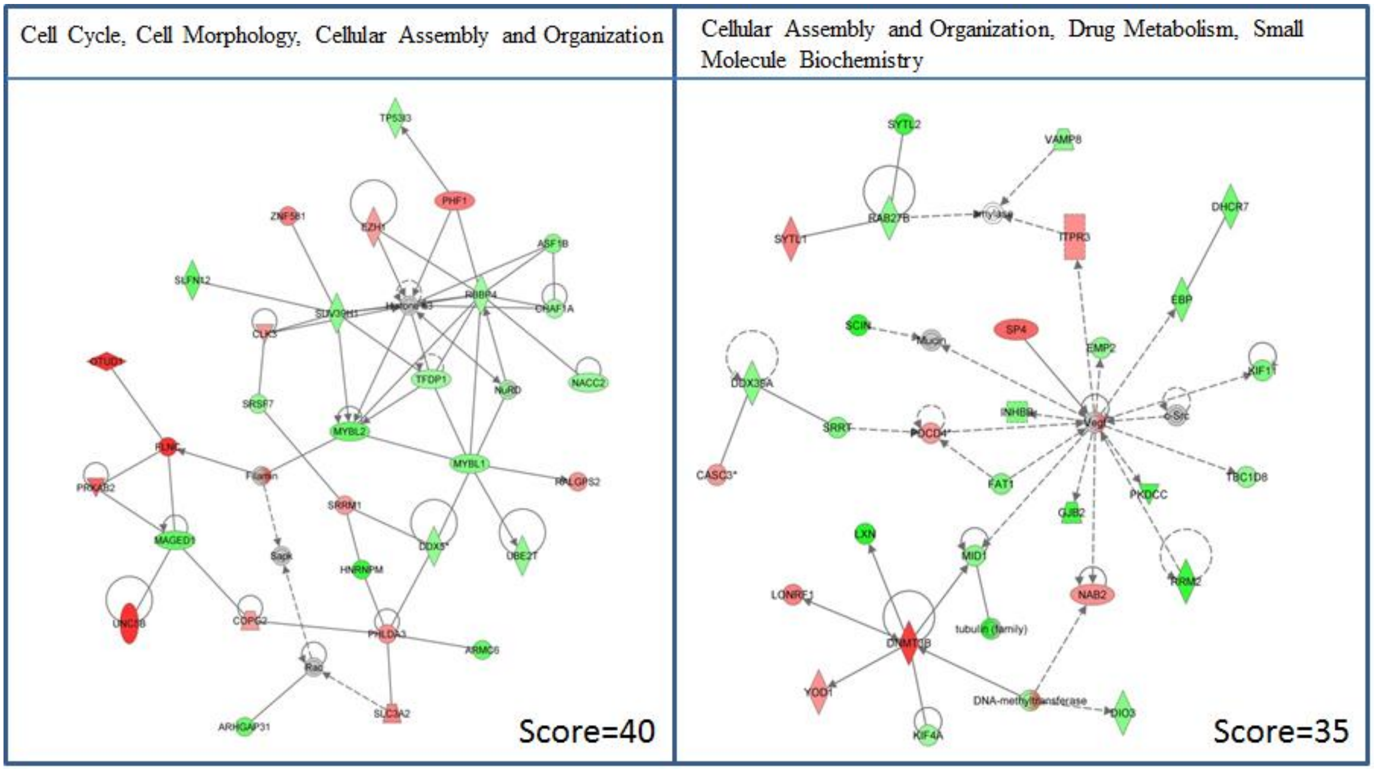

Supplementary Figure S3. Network analysis of genes regulated by the combination of metformin and aspirin using IPA software. The networks with the highest 2 scores (which indicates the statistically significance of the interconnection between the molecules depicted in the network) were shown. The color of the molecules indicates the gene expression (red: upregulation; green: downregulation). Continuous lines represent direct interactions while dashed lines represent indirect interactions.
